# Health related quality of life of people with non-epileptic seizures: The role of socio-demographic characteristics and stigma

**DOI:** 10.1016/j.seizure.2018.01.001

**Published:** 2018-02

**Authors:** Catherine Robson, Lorna Myers, Chrisma Pretorius, Olaug S. Lian, Markus Reuber

**Affiliations:** a Department of Community Medicine, Faculty of Health Sciences, University of Tromsø – The Arctic University of Norway, N-9037 Tromsø, Norway; b The Northeast Regional Epilepsy Group, 820 Second Avenue, Suite 6C, New York, NY 10017, United States; c Department of Psychology, Stellenbosch University, Stellenbosch 7600, South Africa; d Academic Neurology Unit, University of Sheffield, Royal Hallamshire Hospital, Glossop Road, Sheffield S10 2JF, United Kingdom

**Keywords:** Psychogenic non-epileptic seizures, Non-epileptic attack disorder, Quality of life, Socio-demographic factors, Stigma

## Abstract

**Purpose:**

People with non-epileptic seizures (NES) consistently report poorer Health-Related Quality of Life (HRQoL) than people with epilepsy. Yet, unlike in epilepsy, knowledge of how social factors influence the HRQoL of adults with NES is limited. To add to the evidence base, this study explores the relationship between HRQoL and perceived stigma among adults with NES, and the role of socio-demographic characteristics.

**Methods:**

Data was gathered from a survey of 115 people living with the condition, recruited from online support groups. Participants provided socio-demographic and health-related data and completed a series of questions investigating their HRQoL (QOLIE-31) and stigma perceptions (10-item Epilepsy Stigma Scale).

**Results:**

Participants were found to experience high levels of perceived stigma (median 5.2, mean 4.9). A significant and moderate inverse correlation was observed between HRQoL and stigma (r_s_ − 0.474, p = < 0.001); suggesting higher perceptions of stigma contribute to poorer HRQoL among adults with NES. Stigma perceptions were found to be most strongly associated with the seizure worry (r_s_ = − 0.479), emotional wellbeing (r_s_ = − 0.421), and social functioning (r_s_ = 0.407) HRQoL domains. Participants who reported being in employment or education were found to have significantly better HRQoL than those who were not (p = < 0.001).

**Conclusion:**

More (qualitative and quantitative) research is justified to understand how – and why – those with the condition experience stigmatisation, and the factors that impede and help facilitate the participation of people with NES in education and employment.

## Highlights

• Participants reported high levels of perceived stigma.
• A moderate significant inverse correlation was observed between HRQoL and stigma.
• Stigma was associated with seizure worry, emotional wellbeing and social functioning HRQoL domains.
• Participants in employment or education had significantly better HRQoL than those who were not.

## 1 Introduction

There has been a marked shift in thinking about what health is and how it is measured; with traditional clinical outcomes increasingly giving way to, or used in conjunction with, patient reported outcome measures (PROMs) [1]. Health related Quality of Life (HRQoL), is a multidimensional PROM construct used to assess the perceived impact of health status on quality of life; comprised of physical functioning, emotional status, and social well-being domains [2].

People with non-epileptic seizures (NES), often referred to as psychogenic non-epileptic seizures (PNES) or non-epileptic attack disorder (NEAD), consistently report poorer HRQoL than those with epilepsy [[3], [4], [5]]. A recent systematic review of the literature identified 14 studies arising from ten separate research projects (data collections) that have explored associations between independent factors and HRQoL in this patient group [6].

The evidence available suggests a strong adverse association between psychological factors and the HRQoL of adults with NES. Several studies show depression to be a strong predictor of poorer HRQoL in this patient group [[3], [4], [5], [7], [8], [9], [10], [11], [12], [13], [14], [15]]. Other psychological factors associated with poorer HRQoL in people with NES include the number/severity of mood and emotional complaints [[3], [9], [14], [15]], illness perceptions [16], dissociative experiences [[8], [11]], somatic symptoms [[9], [10], [15]], and escape-avoidance coping strategies [[8], [17]]. Condition-related factors, such as older age of onset [[15], [18]] and experiencing the condition for a shorter period of time [15] have also been shown to adversely affect HRQoL. As with epilepsy patient groups [19], seizure freedom has been shown to be positively associated with HRQoL in patients with NES [20]. However, whereas systematic reviews of the literature have found seizure frequency to a (modest) predictor of HRQoL in adults with epilepsy [19], the same was not found to be true for adults with NES [6].

Yet, as Mitchell and colleagues point out [11], studies that attempt to produce a model to explain the factors that are associated with HRQoL in adults with NES only account for 65% of the variance at best [3]. Our limited understanding of how social factors affect the HRQoL of those living with NES probably contributes to this shortfall. There are significant knowledge gaps in relation to domains such as stigma, employment status, and social and family relations [6].

HRQoL in this patient group has been negatively associated with family roles and affective family involvement subscales using the Family Assessment Device (FAD) [5], suggesting the roles and influence of significant others to be a potentially important predictor of HRQoL for people with NES. There is also some evidence that concerns about relationships with the main caregiver seem to cause more distress in those with NES than patients with epilepsy [21]. We know that the stigma associated with epilepsy is considerable and that it has negative effects on HRQoL [22] – in fact, it may account for more HRQoL variance than clinical outcomes (such as seizure frequency and side-effects of antiepileptic drugs) [23]. However, whereas there is a wealth of research to support the view that the social prognosis of epilepsy is often less good than the clinical one [22], comparatively little research has explored the social impact of NES [24], and none has explored the relationship between stigma and HRQoL in this patient group to date. The only study to have examined the role of socio-demographic variables found no significant correlation between employment status, marital status, having children, religious involvement, and proximity to family and HRQoL [15], but more research is needed to substantiate these findings.

To add to the evidence base, this study seeks to explore the relationship between HRQoL and perceived stigma among adults with NES, and the role of participants’ socio-demographic characteristics. Findings (‘statistical pointers’) will inform an upcoming qualitative study exploring the stigma perceptions of people with the condition, which will include exploring participants written texts about their family relations and the social impact of NES. Taken together, we hope to identify social dynamics that will contribute to larger (multiple regression) studies aiming to produce a model to explain factors affecting the HRQoL of adults with NES.

## 2 Methods

A link to an in-depth (86-item, 233-question) survey comprised of polar, frequency, Likert scales, and open questions was advertised to members of 20 patient and practitioner-led online support groups and websites for people with NES (based in the UK and US; not disclosed for reasons of confidentiality, details available on request). The survey was piloted among 25 people living with the condition. Final survey data were organized around four key themes: 1) the diagnostic journey 2) access to and experience of treatment 3) interactions with healthcare professionals and 4) social support and social stigma. Advertising commenced May 2016 and final data collected from 1 July to 1 October 2016.

To include as many people with NES as possible, the only inclusion criteria were that participants had to be over 18 years of age and had received a diagnosis of NES by a health professional. Participants were advised that we used the term NES throughout the survey to describe diagnoses of psychogenic non-epileptic seizures (PNES), non-epileptic attack disorder (NEAD), and other diagnostic terms sometimes used to describe the condition and symptoms; such as, dissociative, conversion, functional, and pseudo seizures. They were also informed that we used the term seizure throughout the survey, whilst recognising that some people experience non-epileptic events in which they do not exhibit movements, only briefly lose consciousness, or experience an altered state of consciousness, or a mixture of these behaviours and sensations. Participants were advised that, unless otherwise stated, to consider the term “seizure” to include such “events”. Those with a dual-diagnosis of epilepsy and NES were asked to only comment on non-epileptic seizures and events wherever possible. Participants were able to save their answers and return to the survey via a secure and automated email link. Typically, open (free-text) questions were optional and all others mandatory. The smart-logic survey format helped to protect against participants giving conflicting answers, and to ‘re-check’ and correct responses when they did so.

This study uses a subset of the full survey data to explore associations between the socio-demographic and health-related characteristics of participants, their HRQoL, and levels of perceived stigmatisation; using the measures listed below.

### 2.1 Measures

Participants were asked a range of socio-demographic and health-related questions, as indicated in Table 1.

**Table 1:** Socio-demographic and health characteristics.

		Number or median	Proportion or range
Country	UK (63) and Ireland (3)	66	57%
	US (42) and Canada (2)	44	38%
	Rest of the world (Australia and Norway)	5	4%
Age		37 years	18–75 years
Gender	Female	102	89%
	Male	11	10%
	Transgender	2	2%
Relationship status	Married/Civil Union (54) or partnered (17)	71	62%
	Single (34) or separated or divorced (10)	44	38%
Living arrangements	Living alone	13	11%
	Living with others	102	89%
Employment status	In full-time (14) or part-time employment (14) or education (9)	37	32%
	Unable to work (72) or home-maker (3)	75	65%
	Retired	3	3%
Disability benefits	In receipt of disability benefits	62	54%
	Not in receipt of disability benefits	53	46%
Time from onset (of NES)		4–5 years	<1 year to 20+ years
Diagnosed by (multiple answers possible)	A neurologist who specialises in seizures	81	70%
	A neurologist who does not specialise in seizures	27	23%
	A psychiatrist or clinical/neuro psychologist	28	24%
Time to diagnosis (of NES)		3–4 years	<1 year to 20+ years
Tests used to diagnose NES (multiple answers possible)	Electroencephalography (EEG)	96	83%
	Ambulatory Electroencephalography (Amb-EEG)	23	20%
	Video-Electroencephalography monitoring (vEEG)	60	52%
	Electrocardiography (ECG or EKG)	69	60%
	Magnetic resonance imaging (MRI)	88	77%
	Computed Tomography (CT/CAT scan)	75	65%
	Tilt table test	15	13%
Prior erroneous diagnosis of epilepsy	Yes	28	24%
	No	84	73%
Self-reported seizure diagnosis	NES alone	100	87%
	NES and Epilepsy	15	13%
NES frequency past month prior to testing		15	0-309

The 31-item Quality of Life in Epilepsy inventory (QOLIE-31) [25] was used to measure HRQoL. The inventory, designed for adults with epilepsy aged 18 years and older, is divided into seven subscales that explore various aspects of patients’ health and wellbeing: emotional well-being, social functioning, energy/fatigue, cognitive functioning, seizure worry, medication effects, and overall quality of life (a single-item subscale). A weighted average of the multi-item scale scores is used to obtain a total score. Although specifically designed for people with epilepsy, there are important clinical similarities and shared concerns between NES and epilepsy patient populations. A review of health status measures did not produce any better tools to assess the construct of HRQoL in this patient population [26]; and a recent systematic review identified the QOLIE-31 as the most popular measure in studies exploring the HRQoL of this patient group [6].

Stigma was measured using the Epilepsy Stigma Scale developed by Dilorio and colleagues [27]. The ten-item scale assesses the degree to which a person believes that their seizure condition is perceived as negative and interferes with relationships with others, rated on a 7-point scale from strongly disagree (1) to strongly agree (7). Item responses are summed to yield a total score. In this study, overall median scores (1–7) were calculated. Higher scores are associated with greater perceptions of stigma. To our knowledge, the measure has not been validated in a NES patient population. We assessed the scale for internal consistency and found α coefficient for the responses of our (n = 115) participants to be 0.89.

### 2.2 Statistical analysis

Analysis of the data was performed using SPSS, version 24. To guard against assumptions of normality and homogeneity of variance, and because measures include ordinal data, non-parametric tests of significance and correlation were used. In some cases, mean scores are presented or discussed for comparative purposes. The primary outcome measure was QOLIE-31 (weighted) total score. The Mann-Whitney U Test was used to compare quantitative variables between two independent groups. Spearman's rank correlation coefficient (r_s_) was used to compare continuous and ordinal data variables. The strength of correlations were defined as: 0–0.39 weak, 0.4–0.69 moderate, and 0.7–1 strong. The coefficient of determination (r_s_^2^) was calculated to establish the proportion of shared variance between HRQoL domains and total stigma score. Holm’s Sequential Bonferroni Procedure [28] was performed for multiple tests to protect against inflation of Type 1 error. Statistically significant results (p < 0.05) not rejected following the Holm’s Sequential Bonferroni method are shown in bold.

## 3 Results

### 3.1 Participants

289 people began the survey. Of these, 141 (49%) completed all mandatory questions and submitted their responses for inclusion in the study. Six people reported a diagnosis other than NES (functional movement disorder) and their responses were excluded from further analysis. Of the remaining 135 participants, we report on 115 participants who described receiving “a formal (highly likely or certain) diagnosis of NES” by a health professional. 20 participants who reported receiving “a tentative (possible or likely) diagnosis of NES” (or who indicated they were awaiting further tests) were excluded from further analysis.

The socio-demographic and health characteristics of the 115 participants included in this study are shown in Table 1.

### 3.2 Health-related quality of life

As a group, participants demonstrated a total median (weighted) QOLIE-31 score of 31.7 (mean 33.8, 95%CI = 31.0–36.7). No significant differences in HRQoL (QOLIE-31 total scores) were observed between those who self-reported a dual diagnosis of epilepsy and NES (median 31.1, range 13.7–63.5), and those with NES alone (median 31.7, range 3.4–87.8) (p = 0.800); nor were significant differences in individual HRQoL domain scores observed between the two groups. No significant associations were found between HRQoL total scores and time from onset of NES (r_s_ = − 0.111, p = 0.239) or time from onset to diagnosis (r_s_ = 0.066, p = 0.486). No significant differences in HRQoL were observed between those who had been erroneously diagnosed with epilepsy in the past and those who had not (p = 0.502). Seizure frequency was shown to be significantly, but weakly correlated with HRQoL (r_s_ = − 0.382, p = < 0.001). HRQoL was not significantly correlated with participants’ age (r_s_ = 0.062, p = 0.512). Following the Holm–Bonferroni method, participants in work or education reported significantly better HRQoL than those who were not. As shown in Table 2, no other socio-demographic variables tested returned a significant result.

**Table 2:** Differences in HRQoL and Stigma between socio-demographic groups.

		QOLIE-31 total score		Stigma Scale total score	
Grouping variable	Groups	Median (and range)	Sig.	Median (and range)	Sig.
Countries	UK and Ireland	33.3 (13.4–87.8)	0.534	5.1 (1.3–7.0)	0.250
	US and Canada	30.6 (3.4–60.2)		5.3 (1.0–7.0)	
Gender	Male	32.0 (11.7–57.0)	0.532	5.0 (3.6–6.4)	0.934
	Female	31.7 (3.4–87.2)		5.2 (1.0–7.0)	
Relationship status	Single, separated, divorced	32.5 (12.6–87.8)	0.929	5.5 (1.3–7.0)	0.022
	Married, Civil Union, partnered	31.1 (3.4–66.5)		5.0 (1.0–6.8)	
Living arrangements	Living alone	39.3 (15.7–87.8)	0.126	5.5 (1.3–6.5)	0.463
	Living with others	30.4 (3.4–66.5)		5.2 (1.0–7.0)	
Employment status	Not in work or education	28.0 (3.4–66.5)	**<0.001**	5.3 (1.0–7.0)	0.017
	In work or education	41.5 (22.2–87.8)		4.8 (1.3–6.6)	
Disability benefits	In receipt	29.6 (9.7–66.5)	0.025	5.3 (1.7–7.0)	0.057
	Not in receipt	37.9 (3.4–87.8)		4.9 (1.0–6.5)	

### 3.3 Stigma

The median stigma score across the whole group of participants was 5.2 (mean 4.9, 95%CI = 4.7–5.2). No significant differences in perceived stigma (total stigma scores) were observed between those who self-reported a dual diagnosis of epilepsy and NES (median 5.2, range 2.7–7), and those with NES alone (median 5.2, range 1–7) (p = 0.718). No significant associations were found between total stigma scores and time from onset of NES (r_s_ = 0.070, p = 0.456) or time from onset to diagnosis (r_s_ = 0.049, p = 0.604). No significant differences in stigma scores were observed between those who had been erroneously diagnosed with epilepsy in the past and those who had not (p = 0.561). Seizure frequency was shown to be significantly, but weakly correlated with perceived stigma scores (r_s_ = − 0.252, p = 0.007). After Holm-Bonferroni correction, no significant differences were observed between the stigma perceptions of socio-demographic groups detailed in Table 2, nor was stigma significantly correlated with participant age (r_s_ = − 0.007, p = 0.942).

A significant and moderate inverse correlation was found between perceived stigma scale and HRQoL (QOLIE-31) total scores (r_s_ = − 0.474, p < 0.001). As detailed in Table 3, analysis of QOLIE-31 subscales (post Holm–Bonferroni method) shows that seizure worry^a^, emotional wellbeing^b^, social functioning^c^ and stigma scale scores were significantly and moderately correlated; the proportion of shared variance for these subscales was ^a^23%, ^b^18%, and ^c^17%. Energy/fatigue and cognitive subscales were found to be significantly, but weakly correlated with stigma. Medicat ion effects and (the single-item) Overall QoL subscales were not found to be significantly correlated.

**Table 3:** Correlations between HRQoL (QOLIE-31) subscales and Stigma Scale scores.

QOLIE-31 subscale scores (weighted)	**r_s_**	**p**
Seizure worry	−**0.479**	**<0.001**
Emotional wellbeing	−**0.421**	**<0.001**
Social functioning	−**0.407**	**<0.001**
Cognitive	−**0.314**	**0.001**
Energy and fatigue	−**0.252**	**0.007**
Medication effects	-0.146	0.120
Overall QoL	-0.132	0.161

## 4 Discussion

This study sought to explore the relationship between social factors (socio-demographic characteristics and stigma perceptions) and the HRQoL of adults with NES. Participants were found to experience high levels of perceived stigma which was inversely correlated with HRQoL. Stigma perceptions were most strongly associated with the HRQoL domains seizure worry, emotional wellbeing, and social functioning. HRQoL was better amongst those in employment or education than those who were not.

The levels of perceived stigma reported by our participants (mean 4.9) are considerably higher than typically found in epilepsy patient populations. A study of 314 people with epilepsy using the same measure reports a mean score of 3.7 [27]. Similarly, a recent study using a single four-point Likert scale question taken from the NEWQOL-6D (*How much do you feel people treat you as an inferior person?*), found that perceived stigma was significantly higher among individuals with NES compared to those with epilepsy [29]. These findings fit with the wider literature, which suggests that people with functional somatic syndromes experience greater perceived stigmatisation than those with comparable organic disease [30].

The stigma of epilepsy is widely reported, and is consistently linked to reduced HRQoL [[22], [31]]. To our knowledge, ours is the first study to explore associations between HRQoL and stigma among adults with NES. A significant and moderate inverse correlation was observed; suggesting higher perceptions of stigma contribute to poorer HRQoL among those with the condition. Stigma perceptions were found to be most strongly associated with seizure worry, emotional wellbeing, and social functioning HRQoL domains; with over one-half of the variability related to these features. There is a dearth of research exploring the social stigma of NES, but peripheral findings from previous studies broadly corroborate our findings. Studies show that people with NES can experience feelings of shame [32], blame and stigmatisation [[33], [34]]; and might conceal the condition and isolate themselves to avoid potential adverse social reactions to seizures and feelings of embarrassment [[34], [35]]. On-going support from family, friends and colleagues has been described extremely important in counteracting the social isolation associated with NES [36].

For people with NES, their stigma perceptions are probably not without foundation. In Western nations derogatory views of NES may be linked to the disparaging use of terms such as ‘psychosomatic’ in the media, which might be taken to mean an illness that is feigned, malingered or representative of a character flaw [37]. Unfortunately, these pejorative opinions are also found in medical circles [38]. For those with the condition, stigmatising interactions with health professionals are not uncommon [39]. People with NES often report their symptoms are met with disbelief, not taken seriously, and that the legitimacy of the illness is sometimes questioned by clinicians [[32], [33], [35], [38], [39], [40], [41]]; and research exploring health professionals’ views supports these assessments [[42], [43], [44], [45], [46], [47], [48]].

Contrary to previous HRQoL findings [15], we found participants who reported being in employment or education (part-time or full-time) to have significantly better HRQoL than those who were not. This discrepancy might be explained the classification of those in education as ‘employed’ in our analysis. Before applying the Holm–Bonferroni method, we also found receipt of disability benefits to be a differentiating factor. As previously observed [15], we did not find relationship status or participants’ age to be discriminating factors. Nor were significant differences observed in relation to participants’ country of residence, gender, or living arrangements and their HRQoL. To our knowledge, these are novel findings and require substantiation.

Seizure frequency was shown to be significantly, but weakly correlated with HRQoL. This finding is in contrast to those of a systematic review which concluded that seizure frequency is not a predictor of HRQoL in this patient group [6]; but is consistent with a study of 96 patients with NES, which found seizure frequency to be significantly associated with lower HRQoL summary scores (SF-36) [10].

Participants’ socio-demographic characteristics were not found to determine stigma perceptions. However, significant differences in levels of felt stigma according to relationship and employment status were noted prior to applying the Holm–Bonferroni method; and seizure frequency was shown to be significantly (albeit weakly) correlated with perceived stigma scores. These findings are consistent with studies of epilepsy patient populations [[27], [49]], but require verification in NES patient populations.

### 4.1 Limitations

While our findings offer a novel contribution to the literature, it is important that they are interpreted within the context of their limitations.

Perhaps the greatest concern with the collection of internet-based patient information is the reliability and validity of the data obtained. Yet, recent reviews suggest health data can be collected with equal or even better reliability in Web-based questionnaires compared with traditional approaches [50]. Participants were able to complete the survey over an extended period if they so wished, and survey metrics show that 97% of respondents completed the survey within 82 h (around 3.5 days). The added benefit of time for reflection, the ability to consider and correct information, and the use of validation checks (as used in our survey) has been shown to improve data quality [50]. There are also strong indications that web-based questionnaires are less prone to social desirability bias [[50], [51]]. Studies show that perceived health status data [52] and HRQoL measures [53] can be reliably collected using online methods. However, it is important to note that the standardised measures used in this study have not been tested in internet-based studies, and research is needed to confirm their online reliability. Due to the design of our study, there is no way to assess response rate. Using number of surveys started as a proxy denominator suggests a completion rate of 49%.

Previous studies exploring the HRQoL of adults with NES have recruited participants from inpatient epilepsy monitoring units (EMUs), outpatient neurology settings, psychotherapeutic centres, or a combination of these; and most report diagnoses were established using video-electroencephalography monitoring (video-EEG) [6]. Video-EEG is the best-practice (‘gold standard’) diagnostic method [54]; however, it is expensive and resource limited [55] and may not be feasible because of the low frequency of seizures [56].

Of the participants in this study only half describe undergoing video-EEG monitoring, with the remainder reporting electroencephalography (EEG) or ambulatory-electroencephalography (Amb-EEG) testing. Our approach means that we cannot say to what extent participants met the diagnostic criteria for NES proposed by the PNES Task Force of the International League Against Epilepsy Non-epileptic Seizures Task Force guidelines [[54], [57]]. We must also consider that the diagnosis of NES is notoriously complex and difficult, and some participants may have been misdiagnosed. In view of the uncertainties about the diagnosis inherent in our recruitment method, the inclusion of people with a dual diagnosis of epilepsy and NES (13% of the sample) could also be considered a limitation of this study. However, given that this study was intended to explore the sociological dimension of NES, we thought it was important not to exclude any subgroup of the whole NES patient population. Epilepsy is an important comorbidity of NES and the 13% figure actually places our study well within the prevalence range of comorbid epilepsy which has previously been reported in HRQoL studies of NES patient populations (6–22%) [[9], [11], [20]]. Participants with mixed seizure disorders were encouraged to think about their NES when responding to questions about their seizures, but we acknowledge that we cannot be certain that all respondents were able to distinguish accurately between their epileptic and nonepileptic seizures.

Despite differences in recruitment methods, other socio-demographic and health-related characteristics of our participants are also within the range of those reported in previous studies exploring the HRQoL of people with NES. In terms of age and gender (mean 31–42 years, 69–100% female) [6]; relationship status (58% married or partnered) [[4], [9]]; proportion in education or employment (45–67%) [[4], [8]]; time from onset of NES (median 3–4 years) [[10], [13]] (mean 4.7–8.9 years) [[3], [5], [8], [18]]; time to diagnosis of NES (median 3.5 years) [11]; and frequency of NES in the four weeks prior to testing (median 6–15 seizures) [[4], [5], [10]] mean (10.9 to 23.7) [[3], [12], [20]]. Data pertaining to physical and psychological comorbidities was not within the scope of our analysis, and our sample might differ from those previously described in these respects.

There might also be important differences between people with NES who have access to the Internet and participate in patient support groups, and those who do not. It is also a weakness of the study that the recruitment method did not allow us to recruit a comparison group, and the study is cross-sectional and correlational, which means that results can be bidirectional and should be interpreted with caution. It is possible that changes in social circumstances or status are more relevant to HRQoL and/or stigma than current circumstances – something best explored longitudinally. The correlational nature of our findings means that we cannot say anything about causalities.

## 5 Conclusion

Despite these limitations, the research improves our understanding of how social factors and dynamics might influence the HRQoL of adults with NES. To our knowledge, the study is the largest HRQoL survey of people with NES to date, and the first to explore the relationship between HRQoL and stigma in this patient group. An important finding is that participants experience high levels of perceived stigma, which negatively affects their HRQoL. Our data suggests that not being in employment or education is detrimental to the HRQoL of people with NES.

These exploratory findings serve a heuristic function, in that they identify several issues for further (qualitative) research. Qualitative analysis can help achieve fuller and more complete descriptions of phenomena, help correct interpretation of quantitative results, and provide triangulation [58]. Perceived stigma could be a treatment target, and research is needed to understand how – and why – those with the condition experience stigmatisation; which in our data was most strongly associated with seizure worry, emotional wellbeing, and social functioning HRQoL domains. More research is also needed to understand factors that impede and help facilitate the participation of people with NES in education and employment. These studies could be usefully followed-up by a project that looks specifically at the enacted stigma faced by this patient group.

## Ethical approval

Ethical clearance was approved by Nelson Mandela University Human Research Ethics Committee (NMU-HREC) on 22nd April 2016 (REF:H16-RTI-RCD-002), where the first author was a visiting researcher at the time of data collection. The survey was hosted by a UK-EU data-protection compliant provider. Potential participants were guided through an online study protocol detailing the purpose of the study, and data protection, ethical compliance and complaint procedures. All participants gave informed electronic consent to participate, and did so again to confirm survey completion and authorise submission of their data for use in the research project.

## Competing interests

No competing interests are disclosed.

## Conflict of interest statement

The authors certify that they have NO affiliations with or involvement in any organization or entity with any financial interest (such as honoraria; educational grants; participation in speakers’ bureaus; membership, employment, consultancies, stock ownership, or other equity interest; and expert testimony or patent-licensing arrangements), or non-financial interest (such as personal or professional relationships, affiliations, knowledge or beliefs) in the subject matter or materials discussed in this manuscript.

### Grant information

Funded by Wellcome Trust Grant REF: grant number: 200923.
